# Supplementary material for: An extended phase graph-based framework for DANTE-SPACE simulations including physiological, temporal, and spatial variations
Source: Magn Reson Med. Author manuscript; Available in PMC 2025 Oct 30. (PMC7618303; doi:10.1002/mrm.30071)
Supplement: Supporting Information [file EMS209615-supplement-Supporting_Information.docx]

**Supporting Information for***“An Extended Phase Graph-based framework for DANTE-SPACE simulations including physiological, temporal, and spatial variations”*

Matthijs de Buck, Peter Jezzard, and Aaron Hess


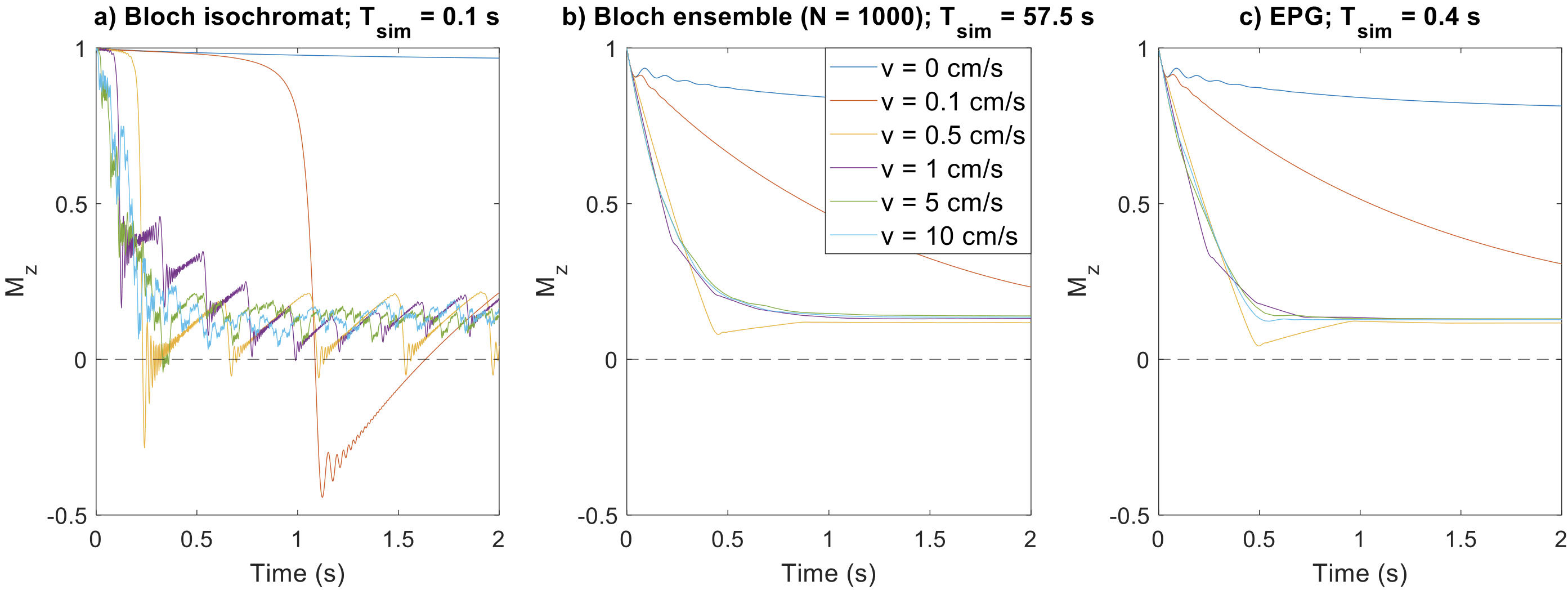


**Supporting Information Figure S1:** Comparison of DANTE simulations using Bloch equations and EPG. The results are shown for **(a)** Bloch isochromat simulations, **(b)** Bloch ensemble simulations, and **(c)** EPG simulations. T_sim_ indicates the computation time of the simulation results shown in each figure.


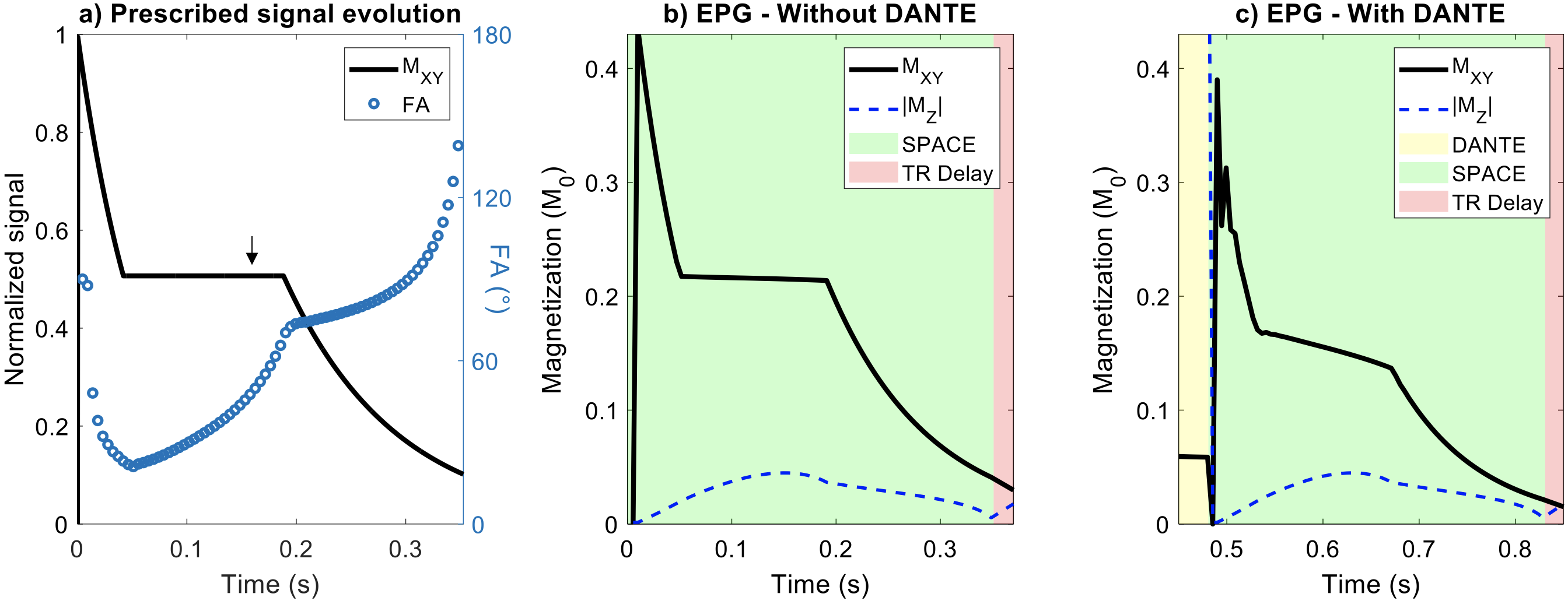


**Supporting Information Figure S2:** Comparison of the transverse magnetization during the SPACE readout. **(a)** shows the prescribed vessel wall signal evolution (black line) for which the SPACE flip angle train (blue circles) is calculated. The black arrow indicates the equivalent echo time of the acquisitions. **(b)** shows the resulting EPG simulation for SPACE acquisitions (without DANTE), while **(c)** shows the EPG simulation for DANTE-SPACE acquisitions. Note that the x-axis in (c) starts near the end of the DANTE preparation.


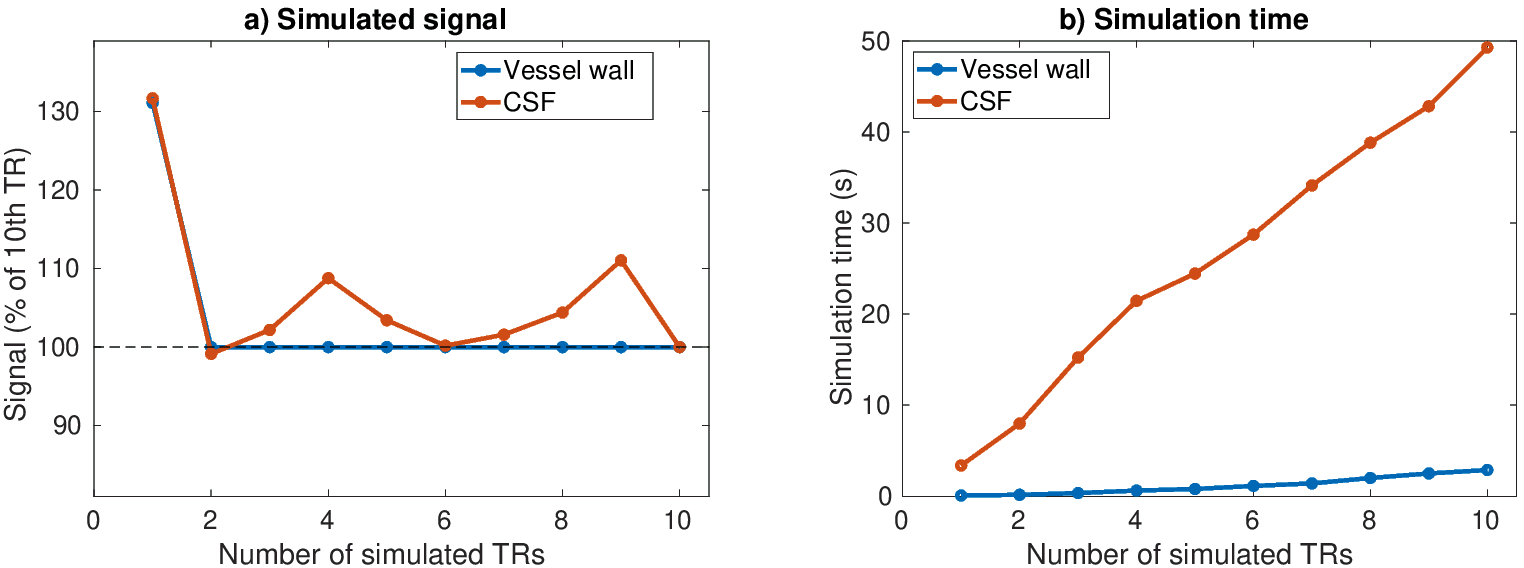


**Supporting Information Figure S3:** Simulation results after increasing numbers of TRs. Results are shown for **(a)** the convergence of the simulated signal and **(b)** the corresponding duration of a single simulation for both (stationary) vessel wall tissue and CSF.


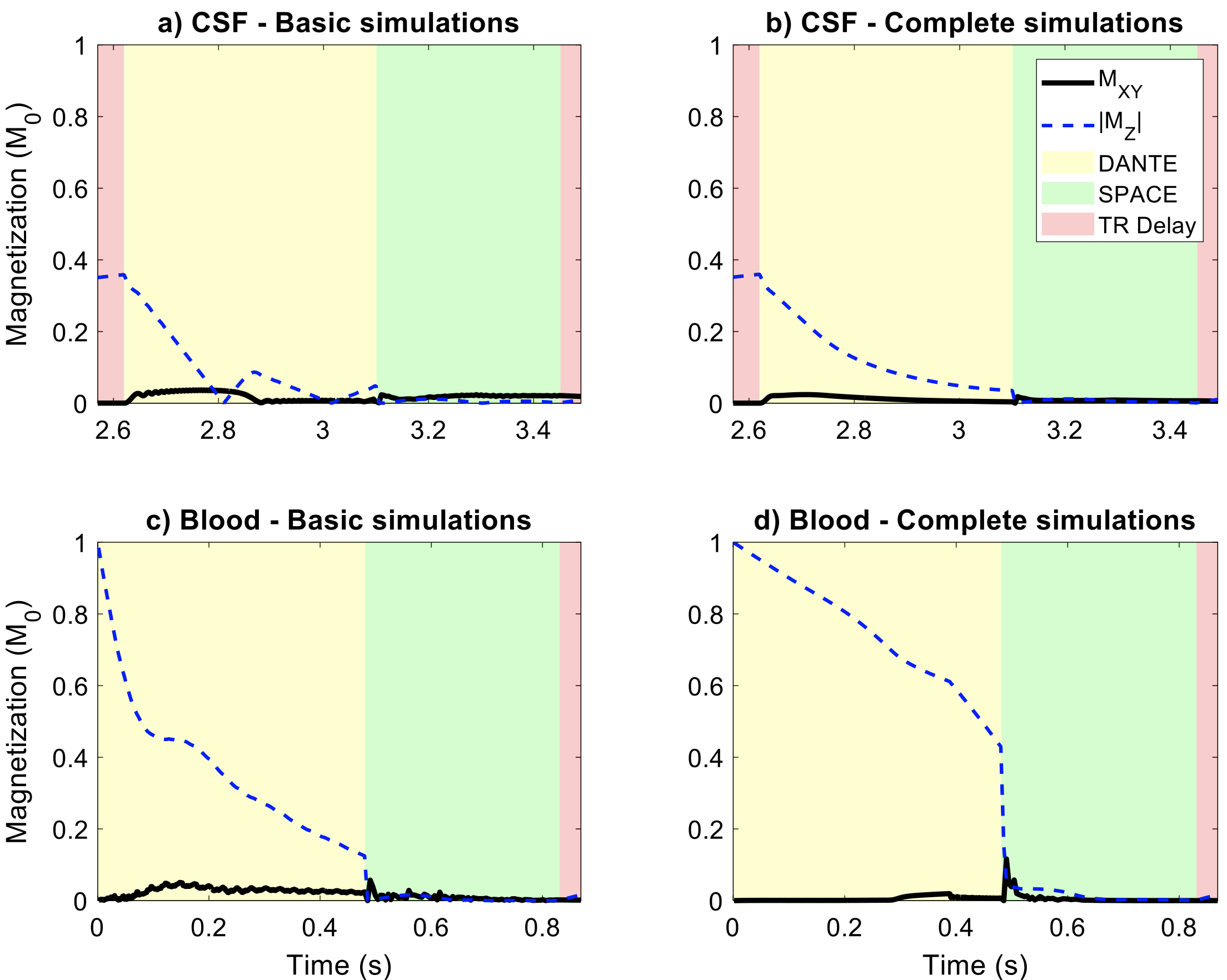


**Supporting Information Figure S4:** The combined effect of intravoxel velocity variation, pulsatile motion, diffusion, flow trajectories, and B1^+^ variation on DANTE-SPACE simulations of CSF (a-b) and blood (c-d), shown for the full y-axis range.

**Supporting Information Figure S5:** Comparison of the signal levels (top row) and signal ratio (bottom row) for T2-weighted DANTE-SPACE protocols using 300 DANTE pulses of 10° (“Protocol 1”) and 200 DANTE pulses of 9° (“Protocol 2”), as described in Section 3.4 of the main manuscript. Results are shown for different levels of simulated B1^+^, diffusion, and vessel wall pulsation velocity, indicating that vessel wall pulsation can explain substantial signal differences between Protocols 1 and 2.


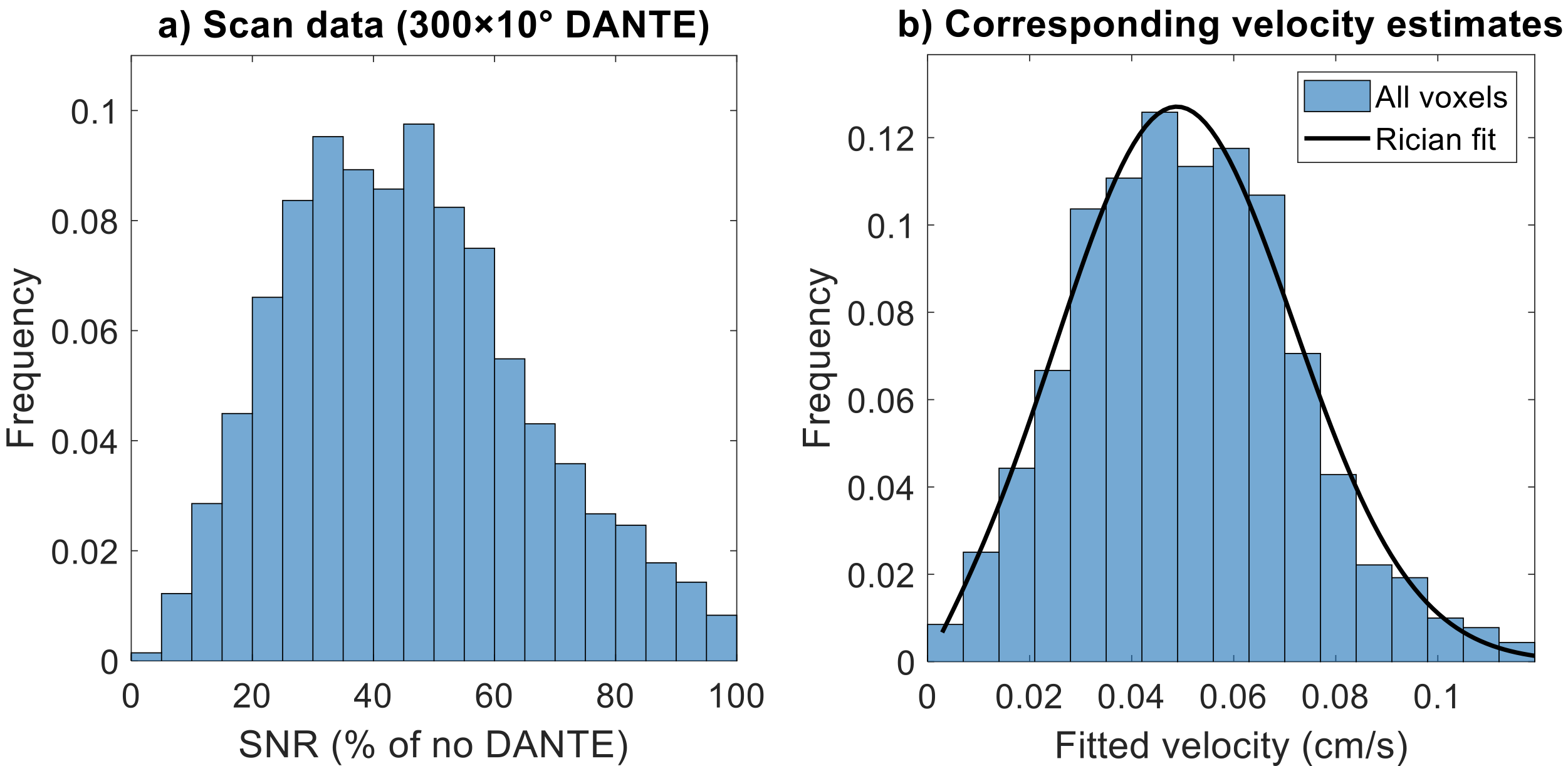


**Supporting Information Figure S6:** Distribution of **(a)** vessel wall signal levels in 5 healthy volunteers in acquisitions using 300 DANTE pulses of 10° (Protocol 1), and **(b)** the corresponding velocity values in simulations. Values in (a) are expressed relative to the 80^th^ percentile of the vessel wall signal in acquisitions without DANTE preparation (using Equation 3 in the main manuscript).


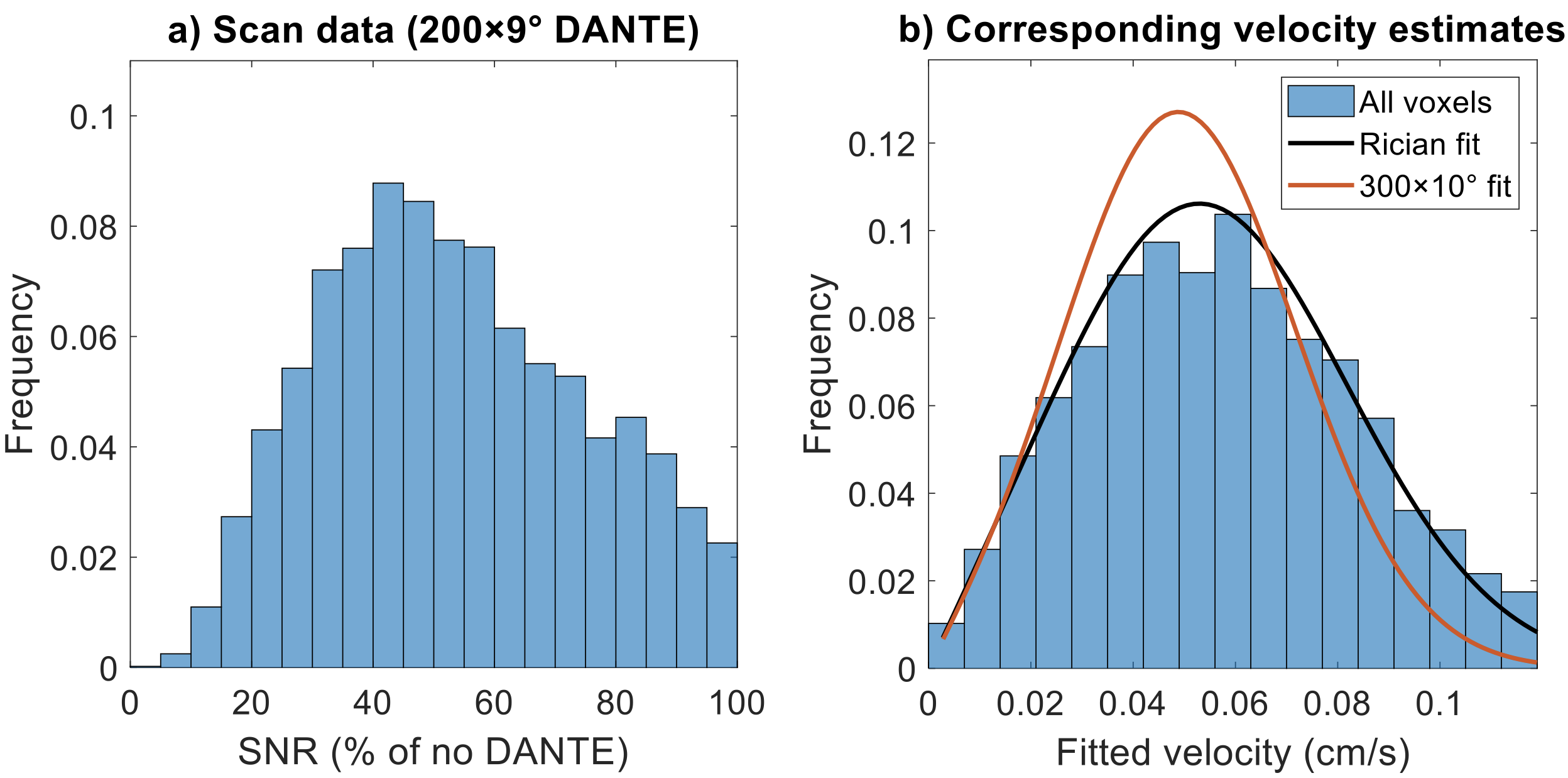


**Supporting Information Figure S7:** Distribution of **(a)** vessel wall signal levels in 5 healthy volunteers in acquisitions using 200 DANTE pulses of 9° (Protocol 2), and **(b)** the corresponding velocity values in simulations. Values in (a) are expressed relative to the 80^th^ percentile of the vessel wall signal in acquisitions without DANTE preparation (using Equation 3 in the main manuscript). The orange curve in (b) shows the Rician fit based on the data in Supporting Information Figure S5 for comparison.

**
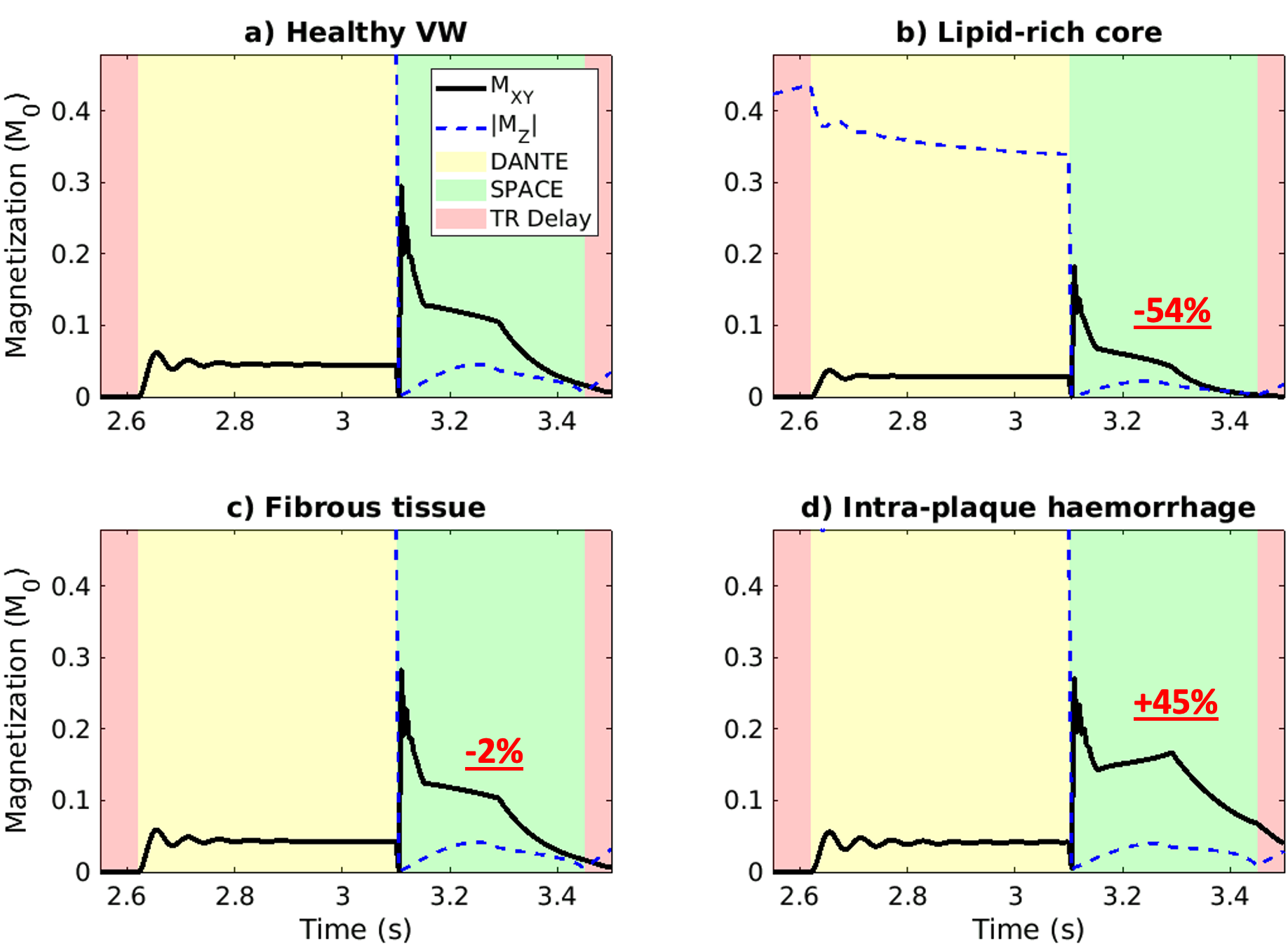
**

**Supporting Information Figure S8:** Comparison of DANTE-SPACE simulations in healthy vessel wall and various plaque components (**(b)** lipid cores, **(c)** fibrous cap tissue, and **(d)** intra-plaque haemorrhages). The sources of the T1 and T2 used for these simulations are described below. The values in red indicate the simulated signal levels in pathology compared to healthy vessel wall tissue.

Relaxation times for plaque components at 7T are available for formalin-fixated *ex vivo* brains^1^, but these have been shown to provide substantially reduced relaxation times compared to the *in vivo* case^2,3^. Therefore, *in vivo* plaque component T2 relaxation times measured at 3T^4^ were scaled based on the T2 of healthy vessel wall at both 3T and 7T^5^ to approximate the expected T2 times at 7T. For T1, *ex vivo* formalin-fixated values^1^ were scaled using the ratio between the T1 of *in vivo*^5^ and formalin-fixated^1^ healthy vessel wall tissue. This resulted in the following T1/T2 estimates at 7T:

- Fibrous cap 1796/47 ms;
- Lipid core 3129/31 ms; and
- Intra-plaque haemorrhage 2290/89 ms.

Note that those values are estimates extrapolated from either *ex vivo* data (for T1) or 3T measurements (for T2). In particular, the relaxation properties of lipid components in atherosclerotic plaques are known to have a temperature dependence due to the differences between liquid and crystallized cholesterols^6^. Since the relevant transition temperatures are around body temperature, this might result in overestimation of the *in vivo* lipid core T1 when calculated from *ex vivo* measurements.

**Additional references for Supplementary Material:**

1. Harteveld AA, Denswil NP, Siero JCW, et al. Quantitative intracranial atherosclerotic plaque characterization at 7T MRI: An ex vivo study with histologic validation. American Journal of Neuroradiology 2016;37:802–810 doi: 10.3174/ajnr.A4628.
2. Lohr D, Terekhov M, Veit F, Schreiber LM. Longitudinal assessment of tissue properties and cardiac diffusion metrics of the ex vivo porcine heart at 7 T: Impact of continuous tissue fixation using formalin. NMR Biomed 2020;33:1–14 doi: 10.1002/nbm.4298.
3. Raman MR, Shu Y, Lesnick TG, Jack CR, Kantarci K. Regional T1 relaxation time constants in Ex vivo human brain: Longitudinal effects of formalin exposure. Magn Reson Med 2017;77:774–778 doi: 10.1002/mrm.26140.
4. Biasiolli L, Lindsay AC, Chai JT, Choudhury RP, Robson MD. In-vivo quantitative T2 mapping of carotid arteries in atherosclerotic patients: Segmentation and T2 measurement of plaque components. Journal of Cardiovascular Magnetic Resonance 2013;15:1–9 doi: 10.1186/1532-429X-15-69.
5. Koning W, de Rotte AAJ, Bluemink JJ, et al. MRI of the carotid artery at 7 Tesla: Quantitative comparison with 3 Tesla. Journal of Magnetic Resonance Imaging 2015;41:773–780 doi: 10.1002/jmri.24601.
6. Yuan C, Petty C, O’Brien KD, Hatsukami TS, Eary JF, Brown BG. In vitro and in situ magnetic resonance imaging signal features of atherosclerotic plaque-associated lipids. Arterioscler Thromb Vasc Biol 1997;17:1496–1503 doi: 10.1161/01.ATV.17.8.1496.
